# EVALUATION OF QUALITY OF LIFE OF PARENTS AND CAREGIVERS OF ASTHMATIC CHILDREN

**DOI:** 10.1590/1984-0462/;2018;36;4;00012

**Published:** 2018

**Authors:** Cristian Roncada, Karina Soldera, Julia Andrade, Luísa Carolina Bischoff, Bianca Martininghi Bugança, Thiago de Araujo Cardoso, Paulo Márcio Pitrez

**Affiliations:** a Pontifícia Universidade Católica do Rio Grande do Sul, Porto Alegre, RS, Brasil.; b Centro Universitário da Serra Gaúcha, Caxias do Sul, RS, Brasil.

**Keywords:** Asthma, Quality of life, Child, Caregiver, Asma, Qualidade de vida, Criança, Cuidador

## Abstract

**Objective:**

To evaluate and compare the levels of quality of life of parents/caregivers of children with and without diagnosis of asthma.

**Methods:**

Parents of children with asthma (asthma group) undergoing outpatient care and parents of children without asthma or asthma in remission (control group) were selected from public schools. They answered a questionnaire about quality of life (The World Health Organization Quality of Life- WHOQOL-BREF), previously validated for the study population. Domains (physical, psychological, social relations, environment and total score) were compared between groups, as well as the levels of correlation of self-perceived quality of life and satisfaction with health.

**Results:**

101 parents/caregivers were included in the sample, that is, 50 (49.5%) parents of asthmatic children and 51 (50.5%) in the control group. Most parents included in the sample were females (n=89; 88.1%), with mean age of 33.5±10.4 years. When assessing quality of life, the overall score of domains was considered satisfactory, both in general evaluation (68.6±13.4) and in each group (asthma: 62.8±10.7; control: 74.3±13.4; p-value<0.001). Comparison of asthma and control groups showed significant differences in total score and in scores of all domains (p<0.001).

**Conclusions:**

Parents/caregiversof children with asthma have a lower quality of life compared to parents/caregivers of healthy children.

## RESUMO

**Objetivo:**

Avaliar e comparar os níveis de qualidade de vida (QV) de pais de crianças com e sem diagnóstico médico de asma.

**Métodos:**

Foi realizado um estudo com pais e cuidadores de crianças com e sem asma no período de 2015 a 2016. Foram selecionados pais de crianças com asma (grupo asma) em acompanhamento ambulatorial e pais de crianças sem asma ou com asma em remissão (grupo controle) em escolas proximais ao estudo, sendo aplicado um questionário respiratório para classificação da amostra. Para avaliação dos níveis de QV, foi aplicado o instrumento desenvolvido pela Organização Mundial da Saúde (OMS)- The World Health Organization Quality of Life (WHOQOL-BREF), previamente validado para a população em estudo, sendo comparados os domínios físico, psicológico, das relações sociais e do meio ambiente e o escore total, além ter sido realizada correlação entre os níveis de autopercepção da QV e da satisfação com a saúde.

**Resultados:**

Participaram do estudo 101 cuidadores de crianças com e sem asma- 50 (49,5%) formaram o grupo asma, e 51 (50,5%), o grupo controle. Amaioria dos genitores é do sexo feminino (n=89; 88,1%), com idade média de 33,5±10,4anos. Naavaliação da QV, o valor do escore total dos domínios foi considerado satisfatório, tanto na avaliação geral (68,6±13,4) quanto por grupos (asma: 62,8±10,7; controle: 74,3±13,4), demonstrando diferenças significativas entre estes em todos domínios estipulados pelo instrumento (p<0,001), bem como no escore total (p<0,001).

**Conclusões:**

Cuidadores de crianças com asma possuem QV significativamente inferior à dos responsáveis por crianças saudáveis.

## INTRODUCTION

Asthma is a chronic, heterogeneous disease characterized by episodic obstruction with hypersensitivity to a wide variety of airway stimuli.^1^ Symptoms may vary from patient to patient and throughout life, and the disease is considered a public health problem frequently affecting the pediatric population. Given its chronicity, orientation of parents or caregivers is indispensable.^2^

According to the Global Initiative for Asthma (GINA), which takes into account symptoms, medications used, activity limitations, lung function and insomnia, asthmatic patients are classified as controlled, partially controlled and uncontrolled, demonstrating that controlling the disease is one of the main forms of treatment. Currently, guidelines are being developed to maintain and expand asthma treatment as a means of controlling it.^3^

Parents’ knowledge about asthma and its triggers is very important, as it may influence treatment adherence and control of symptoms.^4^ The degree of awareness of parents and caregivers may be one of the main causes of demands for emergency care and the rate of hospitalization of children due to asthmatic crises, making the disease control more difficult and impairing quality of life (QoL) levels of both children and their relatives.^5^

Even having levels and variations between people, QoL influences the development or worsening of the disease over time.^6^ Asthmatic children may have problems related to physical and emotional conditions from stress and insomnia.^7^ However, parents can also be affected when attempting to maintain normal day-to-day activities, which compromises the QoL of both.^8^

Asthma can, therefore, be a stressful condition not only to children but also to their caregivers.^9^ The way the family faces illness directly influences children’s adherence to treatment.^10^ Patients’ self-perception of illness, symptoms and psychological/social states affects their QoL and response to treatment.^11^

The aim of this study was to evaluate the QoL levels of parents/caregivers of children diagnosed with asthma by comparing them with a control group and with subgroups, according to the severity of the disease. We also correlated self-perception of QoL and health with physical, psychological, social and environmental conditions and the total score.

## METHOD

To evaluate the QoL of parents/caregivers of children with asthma, a case-control study was carried out with convenience samples, from April 2015 to March 2016.

Parents or caregivers of children with diagnosis of asthma according to GINA criteria^12^ were followed up at a reference center for pediatric asthma in southern Brazil, as well as parents/caregivers of clinically healthy children and children with asthma in remission (asthma episode in the past), selected for convenience in local community schools, as previously applied by the PROASMA Study group,^5^ without episodes of asthma recurrence for at least 12months.

As an inclusion criterion, children diagnosed with asthma should be under ambulatory follow-up for at least 12months. In the group of healthy children or children with asthma in remission, parents could not have direct contact with the disease, such as a second child with asthma or the child with this diagnosis. In the case of the subgroup of patients with asthma in remission, children should be without recurrence of symptoms (asymptomatic) for at least 12months and not under medical supervision or using asthma preventive medicine.

As an exclusion criterion, parents of patients from both groups could not have a diagnosis of asthma or any other chronic disease that could interfere with the study results. In addition, patients could not have another chronic disease that could interfere in the evaluation of the proposed outcome.

For data analysis, patients were divided into asthma group and control group. In addition, subgroups were created for comparison purposes by type and severity of disease

1. control group:
  - healthy, and
  - asthma in remission;
2. asthma group:
  - mild persistent,
  - moderate persistent, and
  - severe persistent.

The sample calculation was made after the inclusion of the first 20 participants, with total QoL score as the main variable of interest. Significance level adopted was 0.05, 90% power, with a standard deviation of 8.2 in the asthma group and 12.5 in the control group, in order to detect a minimum expected difference between averages of 8 points. The size calculated was, therefore, 45 individuals in each group.

To assess QoL, the World Health Organization Quality of Life (WHOQOL-BREF) questionnaire was used,^13^ as validated in Brazilian Portuguese, for parents and caregivers of children diagnosed with asthma in 2015;^14^ the instrument is composed of 26 questions, with answers structured in a 5-point Likert-type scale. The questionnaire was self-administered and focused on respondent’s perception regarding the two weeks prior to the survey. Of 26 questions, two assess self-perceived QoL and health of patients, while all others concern the physical, psychological, social and environmental domains. For the purpose of categorical evaluation of QoL, the WHOQOL-BREF has a cutoff point of ≥60 points for acceptable levels. The closer to 100points, the higher QoL levels of the population studied.^13^ In addition to the WHOQOL-BREF, a general questionnaire, composed of ten questions, was elaborated by the authors to collect data and characterize the sample.

Data were input to a Microsoft Access database (Microsoft Corporation^®^, Redmond, Washington, USA), version 2013, and afterwards exported to the statistical software International Business Machines-Statistical Package for the Social Sciences (IBM SPSS^®^, Nova York, United States), version 20 for Windows. The following tests were performed: Kolmogorov-Smirnov Z to evaluate normality; chi-square to compare categorical data; Student’s t independent test; ANOVA (*post-hoc* Bonferroni test) to compare averages, and Pearson’s correlation between groups and subgroups; finally, a multiple logistic regression was used to evaluate total QoL score according to age of parents, sex, schooling level and number of children. The categorical data are shown in absolute and relative frequencies, and the continuous values are expressed as mean and standard deviation.

The study was approved by the Research Ethics Committee of Universidade Católica do Rio Grande do Sul (PUC-RS), under Supported Opinion No. 379,864. All participants agreed to participate in the study by signing the informed consent form.

## RESULTS

A total of 101 parents/caregivers of children with and without asthma participated in the study; 50 (49.5%) formed the asthma group, and 51 (50.5%), the control group. On asthma severity, 34 (33.7%) participants were non-asthmatic and 17 (15.9%) had asthma in remission, that is, had been asymptomatic for 12months or more (control group); 18 (17.8%) had mild asthma; 20 (19.8%), moderate asthma; and 12 (11.9%), severe asthma (asthma group). Most participants were females (89; 88.1%), with mean age of 33.5 years (±10.4), complete high school [n=26 (25.7%) e n=28 (27.7%), asthma and control groups, respectively] and mean age of children was 1.2 ± 0.8 years, with no difference between groups.

In QoL evaluation (Table 1), measured by the WHOQOL-BREF, mean values were considered satisfactory (cutoff ≥60points) for both total and group assessments, except for the environmental domain for the group of parents of asthmatic children (53.9 ± 14.3). In the comparison between groups, mean values expressed significant differences in all domains established in the instrument and in total score (p<0.001). In the multiple logistic regression test, no explanatory values were found for the dependent variable of total QoL scores and independent factors: parents/caregivers’ age (p=0.631), sex (p=0,438), schooling level (p=0.605), and number of children (p=0.556).

**Table 1:** Assessment of Quality of Life of parents/caregivers of children with and without asthma (asthma group [n=50], control group [n=51] and total [n=101]).

	Asthma	Control	p-value*	Total
	M±SD	M±SD		M±SD
Perception of QoL	70.5±12.0	75.5±19.0	0.119	73.0±16.1
Satisfaction with health	62.5±20.4	74.0±22.3	0.008*	68.3±22.0
Physical domain	61.6±17.8	75.0±14.8	<0.001*	68.4±17.6
Psychological domain	62.6±14.9	75.6±14.5	<0.001*	69.1±16.0
Social relations	64.9±15.9	77.1±16.6	<0.001*	71.1±17.3
Environment	53.9±14.3	68.8±14.3	<0.001*	61.4±16.0
Total score	62.8±10.7	74.3±13.4	<0.001*	68.6±13.4

QoL: quality of life M: mean; SD: standard deviation; *p<0.05.

Upon the evaluation of correlations between the levels of self-perception of QoL and satisfaction with health (Table 2), both groups, showed median/high correlations in all domains and in self-perception total score. The values express a correlation between virtually all scores, except for the asthma group for levels of satisfaction with the disease and with the other domains.

**Table 2:** Pearson’s correlation between levels of perception of quality of life/satisfaction with health related to domains as proposed by The World Health Organization Quality of Life and total score.

Domains	Asthma group				Control group			
	Perception of QoL		Satisfaction with health status		Perception of QoL		Satisfaction with health status	
	r^2^	p-value	r^2^	p-value	r^2^	p-value	r^2^	p-value
Physical	0.37	0.008*	0.18	0.214	0.48	<0.001*	0.44	0.001*
Psychological	0.47	0.001*	0.25	0.083	0.76	<0.001*	0.52	<0.001*
Social relations	0.41	0.003*	0.17	0.228	0.55	<0.001*	0.47	0.001*
Environment	0.49	<0.001*	0.18	0.215	0.75	<0.001*	0.50	<0.001*
Total score	0.72	<0.001*	0.57	<0.001*	0.84	<0.001*	0.75	<0.001*

QoL: quality of life; r^*2*^: Pearson’s correlation; *p<0.05.

**Figure 1:**
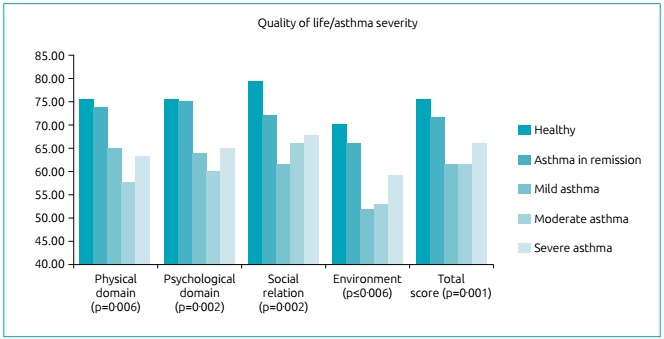
Assessment and comparison of quality of life according to asthma severity levels.

When comparing QoL per disease severity levels, the values indicated differences between parents of children with moderate asthma and asthma in remission (asthma group) or without asthma (control group) for the physical and psychological domains (p=0.006 and p=0.002, respectively). However, for social and environmental domains, as well as for total score, differences were found between the group of children without asthma (control) and patients with mild and moderate asthma (p=0.002; p<0.001, p=0.001, respectively), as shown in Table3 and Figure 1.

**Table 3:** Evaluation and comparison of quality of life according to levels of asthma severity.

	Control group		Asthma group (persistent)			p-value*
	Healthy (n=34)	In remission (n=17)	Mild (n=18)	Moderate (n=20)	Severe (n=12)	
	M±SD	M±SD	M±SD	M±SD	M±SD	
Perception of QoL	77.2±19.8	72.1±17.4	68.1±11.5	70.0±13.1	75.0±10.7	0.230
Satisfaction with health	75.0±22.2	72.1±23.1	59.7±19.4	62.5±23.6	66.7±16.3	0.064
Physical domain	75.6±15.1^†^	73.7±14.6^†^	64.9±16.2	57.5±22.0^‡^	63.7±10.4	0.006*
Psychological domain	75.6±13.4^†^	75.5±16.8^†^	64.3±16.1	59.6±15.9^‡^	64.9±11.4	0.002*
Social relations	79.4±13.6^‡^	72.5±21.0	61.8±14.7^†^	66.2±14.7^†^	67.4±19.9	0.002*
Environment	70.3±13.6^‡^	65.8±15.5^†^	51.7±14.5^†^	52.8±15.5^†^	59.1±11.0	<0.001*
Total score	75.5±12.3^‡^	71.9±15.5	61.8±11.5^†^	61.6±11.9^†^	66.1±10.9	0.001*

M: mean; SD: standard deviation; QoL: quality of life; test applied: Bonferroni post-hoc ANOVA test; ^‡^: main variable presenting statistical significance between variables per Bonferroni’s test ^†^; *p<0.05.

## DISCUSSION

In clinical practice, evaluation of QoL of patients with chronic diseases has gained importance when it comes to patients’’ self-perception, both individually and collectively. With all therapeutic advances, educational measures related to health and environmental hygiene have been shown important in disease control. However, even if patients are able to guarantee a survival to the disease and associated comorbidities, it does not mean that they live “well” or that they “live with quality”, since asthma poses several limitations to children’s daily activities, which, in turn, reflects on their parents or caregivers.

The sample was composed of 101 parents/caregivers of children with or without the pathology, most of them being mothers (caregivers) with mean age of 33.5 years. Similar results have been found in other studies: Fernandes,^15^ using a sample predominantly composed of mothers with mean age of 35 years, and Mendes etal.,^16^ with a population with mean age of 36 years, reported mothers as the main caregivers of these children, reinforcing the idea that caregivers are predominantly young female adults.

Our study found that the QoL of caregivers of children with persistent asthma is lower than that of caregivers of healthy children or children with asthma in remission for all domains addressed by WHOQOL-BREF, in addition to total score (Table 2). Similar data were found by Roncada etal.,^14^ who evaluated the quality of the instrument for this population and described it as presenting good quality of evaluation for the target population and able to identify that parents or caregivers of children have lower QoL levels than parents or caregivers of healthy children.

Another relevant result addressed by the study is the caregivers’ self-perception of their QoL and satisfaction with health. Correlations showed relationships between self-perception of QoL and the values reported for the four domains established by the WHOQOL-BREF, including total score, for both groups (r=0.72 and p<0.001 for the asthma group, r=0.84 and p<0.001 for the control group). As for self-perception of satisfaction with health, the asthma group had correlations shown for total score only (r=0.57, p<0.001), differently from the control group, which presented significant correlations for all four domains and for the total score (r=0.75, p<0.001). These results establish that the samples evaluated have a good perception of their QoL (both groups). However, satisfaction with health is often not considered. These results are similar to Fernandes’,^14^ who reported, after using the WHOQOL-BREF, that 44.9% of caregivers of children and adolescents are satisfied with their QoL, 46.1% reported being quite sure about it and 41.6% consider the option “more or less” to measure how healthy their physical environment is.

QoL per severity of the disease had significant differences between parents of healthy children or children with asthma in remission and other levels of severity (persistent) regarding all four domains (physical, psychological, social and environmental), as well as total QoL score (p=0.006, p=0.002, p=0.002, p<0.001 and p=0.001, respectively). Unexpectedly, parents of children with severe asthma, among all levels of severity, had the most acceptable levels of QoL in comparison to others (mild and moderate asthma). This stems from the fact that these children have severe, therapy-resistant asthma (STRA) and are linked to the specific treatment program with Omalizumab (OMB), which provides better disease control due to the specific treatment applied. In 2015, Sztafinska etal.^17^ published a study with 19 STRA children and evaluated health-related QoL levels of both caregivers and patients pre- and post-treatment with OMB.

It is of utmost importance to carry out further studies on this matter, as the QoL of caregivers of children with asthma may directly or indirectly interfere with the child’s treatment and care.^18^ Patients in this condition need special care, and so it is vital to know how caregivers face this reality.^19^ Even knowing the importance of approaching this subject, many studies^20^ ^,^ ^23^ ^,^ ^24^ focus only on the QoL of children with asthma, not taking into account the subjectivity of the disease and the proportion that morbidity reaches. In addition, based on information from other studies,^25^ ^,^ ^26^ ^,^ ^27^ one recognizes that caregivers also need psychological support because of the attrition caused by the routine related to this disease, which can be harmful to them and to the patients.

According to the results, one can conclude that parents/caregivers of children and adolescents with asthma have lower QoL compared to caregivers of healthy children and adolescents or with asthma in remission. The type of care that the disease requires causes caregivers to feel more exhausted than others, which can directly contribute to their and their dependents’ QoL level.
